# The G6PD flow-cytometric assay is a reliable tool for diagnosis of G6PD deficiency in women and anaemic subjects

**DOI:** 10.1038/s41598-017-10045-2

**Published:** 2017-08-29

**Authors:** Germana Bancone, Michael Kalnoky, Cindy S. Chu, Nongnud Chowwiwat, Maria Kahn, Benoit Malleret, Pornpimon Wilaisrisak, Laurent Rénia, Gonzalo J. Domingo, Francois Nosten

**Affiliations:** 1 Shoklo Malaria Research Unit, Mahidol–Oxford Tropical Medicine Research Unit, Faculty of Tropical Medicine, Mahidol University, Mae Sot, Thailand; 2 Diagnostics Program, PATH, Seattle, USA; 3 Singapore Immunology network (SIgN), A*STAR, 8A Biomedical Grove, Singapore, 138648 Singapore; 4 Department of Microbiology and Immunology, Yong Loo Lin School of Medicine, National University of Singapore, National University Health System, 5 Science Drive 2, Blk MD4, Level 3, Singapore, 117597 Singapore; 5 Centre for Tropical Medicine, Nuffield Department of Medicine, University of Oxford, Oxford, United Kingdom

## Abstract

Glucose-6-phosphate dehydrogenase (G6PD) activity is essential for redox equilibrium of red blood cells (RBCs) and, when compromised, the RBCs are more susceptible to haemolysis. 8-aminoquinolines (primaquine and tafenoquine) are used for the radical curative treatment of *Plasmodium vivax* malaria and can cause haemolysis in G6PD deficient subjects. Haemolytic risk is dependent on treatment dose and patient G6PD status but ultimately it correlates with the number of G6PD deficient RBCs. The G6PD spectrophotometric assay reliably identifies deficient subjects but is less reliable in heterozygous females, especially when other blood conditions are present. In this work we analysed samples with a range of G6PD phenotypes and haematologic conditions from 243 healthy volunteers of Asian or African-American heritage using both the spectrophotomeric assay and the G6PD flow-cytometric assay. Overall 18.5% of subjects (29.3% of Asian females) presented with anaemia, associated with decreased RBCs volume (MCV) and reticulocytosis; the flow-cytometric assay showed good correlation with the spectrophotometric assay (Pearson’s r 0.918–0.957) and was less influenced by haemoglobin concentration, number of RBCs and number of reticulocytes. This resulted in more precise quantification of the number of G6PD deficient RBCs and presumably higher predictive power of drug induced haemolytic risk.

## Introduction

Glucose-6-phosphate dehydrogenase ﻿(G6PD) deficiency is caused by mutations that impair the stability of the protein; the enzymatic capacity of the protein to reduce NADP+ in red blood cells is used, among other biochemical properties, to characterize the deficient variants. The G6PD gene is located on the X-chromosome therefore different genotype and phenotypes are observed among males and females ; males and females can be hemizygous/homozygous for the normal or mutated mutation and fully express the associated normal or deficient phenotype while only in females the heterozygous genotype can be associated with a wide range of phenotypes due to the X-chromosome inactivation^1^. In areas where G6PD deficiency exists, it often reaches a prevalence of 15–20% among males which corresponds to a 25–30% of females with the heterozygous genotype whose phenotypic diagnosis represents a challenge. Owing to the biological phenomenon of X-chromosome inactivation, women with greater than 30% activity, although showing an overall activity higher than what it is considered deficient, may still be at risk for clinically important haemolysis after treatment with oxidant drugs^2^ because a considerable proportion of their RBCs are G6PD depleted. Safe use of antimalarials such as primaquine and tafenoquine for the radical cure of *Plasmodium vivax* in women requires a better understanding of the relationship between G6PD activity and risk of haemolysis in those with an intermediate G6PD phenotype (30–80% normal activity).

The gold standard for G6PD diagnosis at the laboratory level is the spectrophotometric assay^3^ which detects the total enzymatic activity in a haemolysate of blood (which can be depleted of white blood cells for more accurate analysis). Activity is calculated as change in absorbance at 340 nm (ΔAbs) at a given temperature and normalized by either the concentration of haemoglobin (giving a results in unit of enzyme per grams of haemoglobin, IU/gHb) or, more rarely, by the number of RBCs (unit of enzyme per RBC, U/RBC). The technique is influenced by the concentration of haemoglobin in the blood sample and can result in an estimated value of G6PD activity that is falsely high in subjects with anaemia or iron deficiency. Factors such as reduced RBCs volume, increased number of reticulocytes or a combination of both are believed to contribute to the increase in assessed activity.

Cytochemical techniques can detect an active G6PD enzyme at the single red cell level but they are laborious and technically challenging. The methaemoglobin reduction test (MRT) first developed by Brewer^4^ exploits the capacity of RBCs with a functioning G6PD enzyme to reduce oxidized methaemoglobin (MetHb) to normal Hb. The method developed by Fairbanks and Lampe^5^ is based on the capacity of G6PD to reduce [3-(4,5-Dimethylthiazolyl-2)-2,5-Diphenyltetrazolium Bromide] (MTT) into insoluble tetrazolium salts. Stained cells are then graded for the number of intracellular grains (‘dots’) which correlates to intracellular G6PD activity. The cytochemical staining method developed in 1985^6^ is also based on the reduction of tetrazolium salts and it is suitable for analysis by flow-cytometry^7^ but requires several hours of sample processing and staining and is technically challenging. Recently a more simple and rapid flow-cytometric read-out of the MRT has been developed^8, 9^.

In the past, assessment of G6PD activity performed by both cytochemical techniques and biochemical orspectrophotometric assays have shown generally good correlation; the cytochemical techniques showed higher diagnostic power in heterozygous females^10–13^. No specific comparison of diagnostic accuracy between the two techniques has been performed so far in anaemic subjects or carriers of haemoglobinopathies. From a clinical point of view, it is difficult to predict whether chronic anaemia could be associated with an increased haemolytic risk since this would depend on the cause of anaemia (an increased number of reticulocytes due to chronic RBCs destruction seen in the thalassaemias versus bone marrow suppression for example). Whilst it is not expected that subjects with severe G6PD deficiency would be mis-diagnosed even when anaemic, a correct quantitative characterization of phenotype in heterozygous women, including those with anaemia, is essential for the safe deployment of radical curative treatment for *P. vivax* using high dose primaquine or other 8-amoniquinolines. In the present study we analysed samples collected among Karen and Burman people in Thailand and among African-Americans in the USA for a total of 243 healthy male and female volunteers with normal, intermediate and deficient G6PD phenotypes. We carried out complete blood characterization (including haemoglobin typing for the Asian samples), G6PD phenotypic characterization by both spectrophotometric and flow-cytometric assays and genotyping by full gene sequencing. In the study populations a sizable proportion of healthy subjects were expected to be anaemic and/or carriers of haemoglobinopathies therefore it was possible to analyse haematologic factors that may influence quantitative assessment of G6PD enzymatic phenotype with the two methodologies used.

## Results

### G6PD genotyping, haemoglobin type and associated haematologic characteristics

The study population was composed of 146 adult females and 97 adult males. Volunteers were recruited using a targeted enrolment strategy to allow the enrolment of a larger proportion of G6PD deficient subjects and heterozygous females as compared to the general population. Overall 48 G6PD mutated hemizygous males, 18 homozygous females (of whom one with 2 concomitant variants), 90 heterozygous females and 87 wild type (38 women and 49 men) were included (Table S1). Among the heterozygous women, one was carrier of a new variant (701 T > C corresponding to amino acid change Ile234Thr) here named “Shoklo”. Hemizygotes and homozygotes were pooled for analysis and the group was called “hemi/homozygotes”.

Blood features for the all samples are presented in Table 1 according to gender. Significant differences were found, as expected, in the number of RBCs and levels of haemoglobin (Hb), concentration of Hb and haematocrit. Anaemia defined as [Hb] ≤ 11.5 g/dL was found in significantly more women (23.8%) than men (10.1%, P = 0.007).

**Table 1:** Haematologic parameters [Mean (SD)] of study participants according to gender.

Gender	N	WBC (10^3^/µL)	RBC (10^6^/µL)	HGB (g/dL)	HCT (%)	MCV (fL)	PLT (10^3^/µL)
Male	97	6.17 (2.07)	4.85 (0.55)	13.55 (1.60)	41.13 (3.79)	85.24 (6.91)	252.48 (74.25)
Female	146	6.77 (1.92)	4.47 (0.51)	12.43 (1.34)	37.29 (3.76)	83.85 (6.56)	298.23 (80.88)
P value		0.022	8.30E-08	1.01E-08	2.26E-13	0.113	1.26E-05

**Figure 1:**
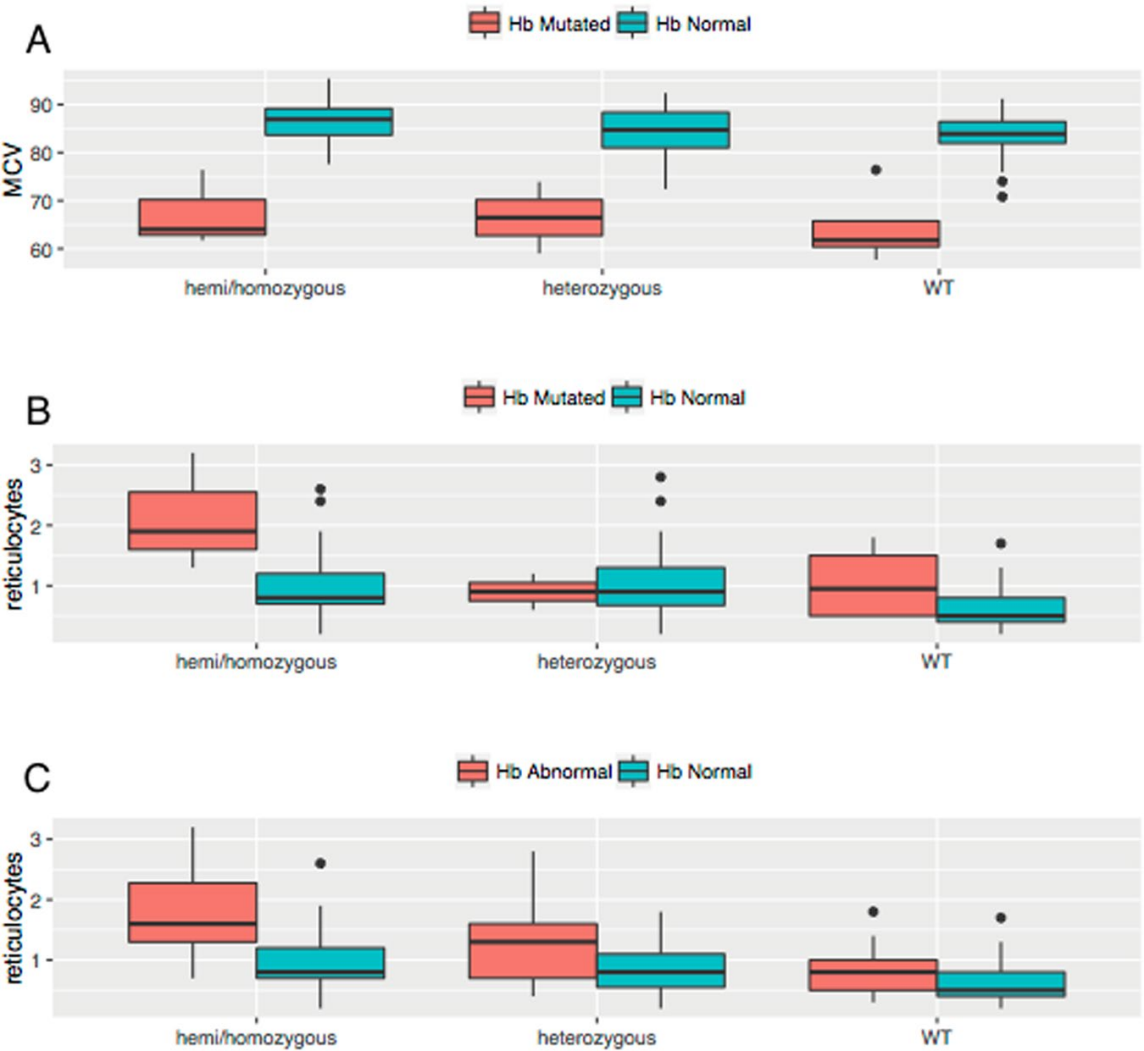
Analysis of MCV and reticulocytes according to G6PD and Hb variants. (**A**) Measured MCV by G6PD genotypes (G6PD hemi/homozygote; G6PD heterozygote; G6PD WT) and Hb type (Hb Normal and Hb mutated); Within the normal Hb group, MCV is statistically higher in G6PD hemi/homozygotes as compare to both heterozygote and wild type. (**B**) Measured reticulocytes by G6PD genotypes (G6PD hemi/homozygote; G6PD heterozygote; G6PD WT) and Hb type (Hb Normal and Hb mutated); within the Hb normal group, reticulocyte count are statistically higher in both G6PD heterozygotes and hemi/homozygotes as compared to G6PD wild type. (**C**) Measured reticulocytes by G6PD genotypes (G6PD hemi/homozygote; G6PD heterozygote; G6PD WT) and anaemia [Hb Normal vs Hb abnormal (Hb mutated or anaemia)]; within the Hb normal group, reticulocyte count are statistically higher in both G6PD heterozygotes and hemi/homozygotes as compared to G6PD wild type. Within the Hb abnormal group, reticulocyte counts are statistically higher in G6PD hemi/homozygotes only when compared to wild type. Statistical significance of all comparisons is summarized in Supplementary Table S3.

In the Asian samples, haemoglobin typing results showed that 10% of subjects had an abnormal haemoglobin type, 7 (4.8%) were beta-thalassaemia carriers, 5 (3.4%) were confirmed or suspected alpha-thalassaemia carriers, 2 (1.4%) were HbE carriers and 1 was Hb Constant-Spring carrier. For the purpose of the analyses, alpha-thalassaemia carriers were pooled with Hb normal (since their haematologic profiles were similar) and all subjects with other Hb variants were pooled together. Sixty percent of subjects with Hb variants were anaemic as compared to 17.9% among the Hb normal group (P < 0.01). The red cells volume (Mean Corpuscolar Volume, MCV) was analysed further according to the Hb type and G6PD genotype (Fig. 1A) since it is known to be low in subjects with Hb variants and has been shown to increase in subjects with G6PD deficient variants^14^. These predictions were confirmed by statistical analysis where MCV values for normal Hb samples in G6PD WT samples were significantly greater than Hb abnormal samples (P < 0.01) and, within the Hb normal group, MCV in G6PD deficient hemi/homozygotes was significantly greater than in G6PD heterozygotes and WT (P = 0.040 and P = 0.005 respectively). In the Asian sample only, reticulocyte count was also analysed according to G6PD genotype and Hb variant (Fig. 1B). It is known that subjects with mutations on the G6PD gene have a higher reticulocyte count compared to G6PD wild type however, it is unclear whether subjects with Hb mutations might have increased reticulocyte count compared to Hb normal subjects. We performed statistical analysis to compare the distribution of reticulocytes in Hb normal specimens with G6PD mutations where hemi/homozygotes and heterozygotes had significantly elevated reticulocyte counts (P = 0.002 and P = 0.0001 respectively). When analysing subjects with Hb abnormal status, defined as having either a mutated Hb or being anemic (Fig. 1C), elevated reticulocytes were found in G6PD hemi/homozygotes only when compared to wild type (P = 0.028). Overall the data confirmed that carriers of abnormal haemoglobins had decreased haemoglobin concentration and smaller MCV as compared to Hb normals, while subjects with G6PD deficiency, carriers of haemoglobinopathies or anaemic had higher reticulocyte number.

### G6PD phenotypes

**Figure 2:**
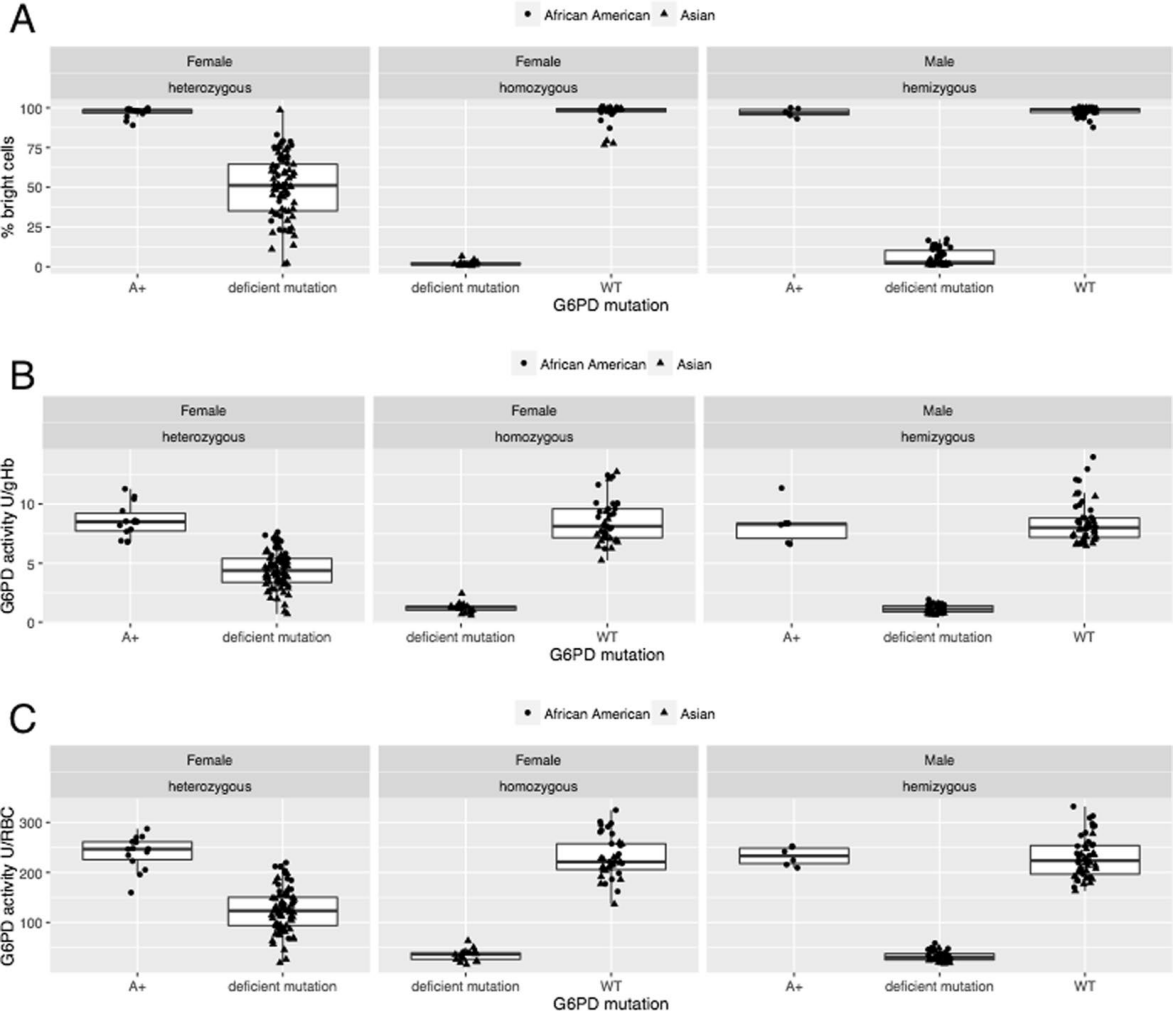
G6PD activity assessed by flow-cytometry (**A**) and spectrophotometry (**B** and **C**). Percent bright cells (G6PD normal), G6PD activity IU/gHb and G6PD activity U/RBC distributions by gender and G6PD mutations. Box plots for the distributions of % percent bright cells observed per specimen per G6PD genotype are shown highlighting minimum and maximum (whiskers), 1^st^ quartile and 3^rd^ quartiles (boxes), and means. “Deficient mutations” includes the following G6PD variants: A-, Mahidol, Viangchan, Kaiping, Shoklo, Orissa.

In the Asian samples, G6PD results generated by flow-cytometry were analysed by both FlowJo software and a recently developed web-based tool called Mosaic G6PD Flow Tool; results from the new software were in excellent agreement (R^2^ = 0.995) with the standard analysis therefore in this manuscript all results obtained using the Mosaic G6PD Flow Tool were used. Figure 2 shows the distributions of G6PD phenotypes according to the genotypes: by flow-cytometry as percentage of RBC with normal G6PD activity (A) and by spectrophotometry calculated as IU/gHb and IU/RBC (B and C). As expected, in both assays, the heterozygous women showed a wide distribution of activities while homozygous genotypes (both mutated and wild type) were at the extremes of the distribution. In the flow-cytometry method, the mutated hemi/homozygous genotypes always showed <10% normal RBCs. Conversely the wild type genotypes showed at least 85% G6PD normal RBCs giving rise to extremely clear phenotypes. Assessment of activity by spectrophotometry showed instead larger distributions of phenotypes also in hemi/homozygous genotypes.

**Figure 3:**
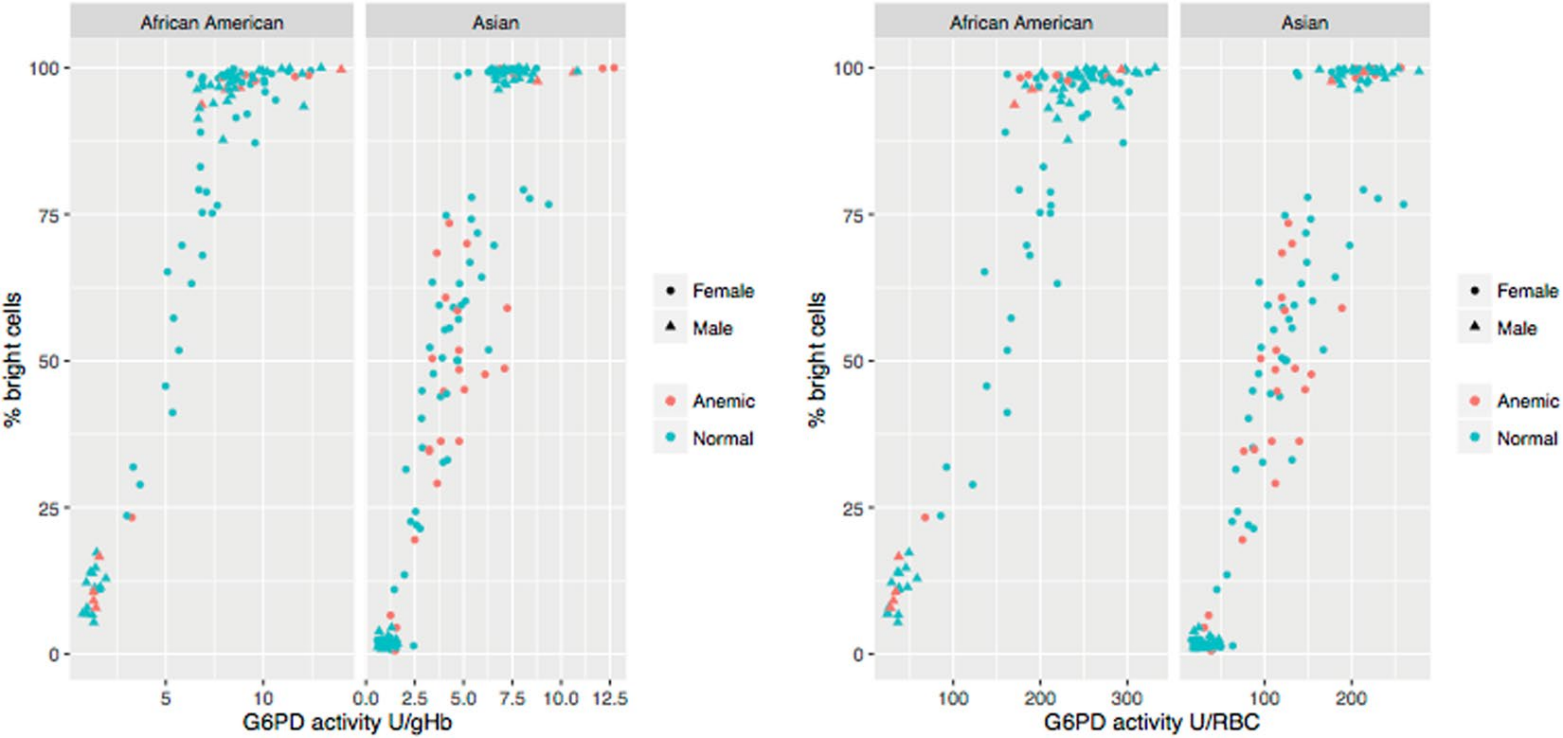
Correlation of G6PD activity assessed through spectrophotometric and flow-cytometric assays. Red dots are subjects with anaemia or Hb abnormal type. [All samples] Spectrophotometric (IU/gHb) and flow-cytometry: Pearson’s r = 0.925 (Asian), r = 0.915 (A-American) and r = 0.917 (pooled). Spectrophotometric (U/RBC) and flow-cytometry: Pearson’s r = 0.949 (Asian), r = 0.925 (A-American) and r = 0.932 (pooled). [Subjects with anaemia or Hb abnormal excluded] Spectrophotometric (IU/gHb) and flow-cytometry: Pearson’s r = 0.957 (Asian), r = 0.918 (A-American) and r = 0.930 (pooled). Spectrophotometric (U/RBC) and flow-cytometry: Pearson’s r = 0.955 (Asian), r = 0.925 (A-American) and r = 0.934 (pooled).

The correlation between the two assays was analysed by comparing the spectrophotometric enzymatic activity to the percentage of G6PD-normal RBCs (assessed by flow-cytometry) among all samples (Fig. 3A,B). The correlation was good when spectrophotometric activity was expressed as IU/gHb (Pearson’s r = 0.925, r = 0.915 and r = 0.917 in the Asian, African-American and pooled samples respectively) and very good when spectrophotometric activity was expressed as U/RBC (r = 0.949, r = 0.925 and r = 0.932 in the Asian, African-American and pooled samples respectively). All P values were < 2.2e-16.

When subjects with anaemia and abnormal haemoglobin type were excluded from the analyses, even higher correlations between the two methods were found when activity was expressed as IU/gHb (r = 0.957, r = 0.918 and r = 0.930 in the Asian, African-American and pooled samples respectively). No difference was seen from the analysis with the full sample when activity was expressed as U/RBC (r = 0.955, r = 0.925 and r = 0.934 in the Asian, African-American and pooled samples respectively). All P values were < 2.2e-16.

### Comparison of activity assessed by spectrophotometry and flow-cytometry in heterozygous women

**Figure 4:**
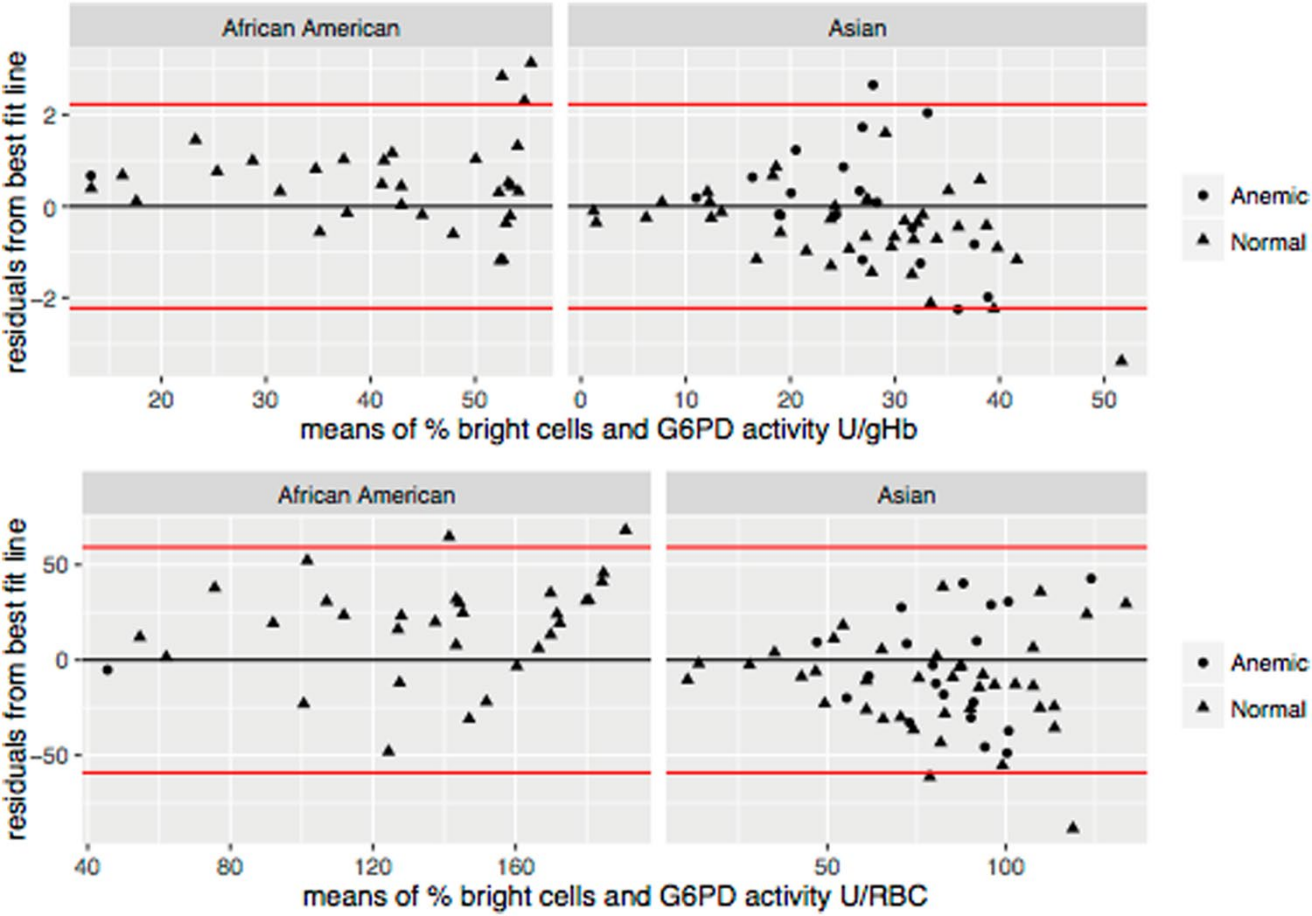
Bland-Altman plot for comparison of spectrophotometric and flow-cytometric assays in heterozygous women. Black lines are the mean of residuals for the best fit line between flow-cytometry and spectrophotometry for all heterozygous samples and red lines are two standard deviations of the residuals from the mean or best fit line between the two assays. Plots are faceted by Asian and African-American samples; subjects with anaemia and/or abnormal Hb type are denoted by the circle while subjects with normal Hb type and normal Hb concentration are denoted with the triangle.

Inaccurate diagnosis of G6PD status is more likely in heterozygous women; using the data from 88 heterozygous women we compared the quantitative assessment of G6PD activity between spectrophotometry and flow-cytometry. The spectrophotometric activity as IU/gHb and U/RBC was compared with the actual percentage of normal RBCs (i.e. percent bright cells) as counted by the flow cytometer using a Bland–Altman plot (Fig. 4). On average there was a small difference between the two techniques and the variance around the mean difference increased with G6PD activity expressed in IU/gHb.

## Discussion

In the present study we compared G6PD activity assessed by the flow-cytometric assay to the gold standard spectrophotometric assay in a large number of healthy subjects from two different populations presenting a wide range of G6PD activities and haematologic pictures.

The data showed that the flow-cytometric assay can be used to assess the actual number of RBCs with and without G6PD activity in freshly collected blood samples. Recently Kahn and colleagues^15^ have shown that the assay can be used in cryopreserved RBCs and in blood stored for a few days (provided that they are supplied with additional substrate). In the current study we have tested stability of samples after staining (Supplementary Figure S1, Supplementary Figure S2 and Supplementary Table S2) and we have shown that G6PD deficient RBCs tend to haemolyse after just one hour post-staining. Therefore flow-cytometric analysis should be performed within one hour otherwise the relative proportion of normal and deficient red blood cells can drastically change over time, especially in blood from heterozygous women.

Previous studies have already compared the performances of flow-cytometric technique against spectrophotometric assay in blood with African G6PD variants^8, 9^ showing very good correlation overall. In this study we confirm those findings and highlight the differences observed when activity is calculated with respect to Hb concentration or number of RBCs in blood samples coming from subjects with anaemia and other blood conditions. Anaemia was found in 18.5% of the overall sample and as high as 29.3% in females in the Asian samples; indeed, this represents a sizeable proportion of the population where oxidative anti-malaria treatments are expected to be deployed. The correlation between flow-cytometry and spectrophotometry was very good in non anaemic, Hb normal subjects; in the whole sample the concordance between the two techniques was higher when G6PD activity was expressed as U/RBC rather than IU/gHb (Fig. 3). The lower correlation found between spectrophotometry (IU/gHb) and flow-cytometry in anaemic subjects could be due to a mere numerical issue, i.e. the absorbance value is normalized by a low numeric value of Hb giving a falsely high result when activity is expressed as U/gHb. From our data the increased number of reticulocytes in anaemic subjects seemed to play a role. Reticulocytes are likely to increase the overall spectrophotometric activity in hemolysate due to their larger size and higher active metabolisms^16, 17^ while with the flow-cytometric technique they are classified just as G6PD normal RBCs. In anaemic subjects thus the spectrophotometric assay tends to overestimate the proportion of G6PD normal RBCs (resistant to haemolysis).

When anaemia is caused by abnormal Hb variants, questions could be raised about the haemolytic risk associated with 8-aminoquinoline treatments in carriers of both Hb variants and G6PD mutations. Distribution of abnormal haemoglobin variants is super-imposable on that of G6PD deficiency and malaria^18^; in certain populations of Southeast Asia, Hb E prevalence reaches peaks of 60–70%^19^. Both Hb E and beta thalassaemia have been found to be associated with increased intracellular oxidative stress^12, 20, 21^. In contrast in early observations of the Sardinian population where beta-thalassaemia and G6PD deficiency co-exist, it was noted that subjects with G6PD deficiency and concomitant beta-thalassaemic trait were less likely to have favism as compared to G6PD deficient subjects with a normal haemoglobin type^22, 23^. Other early reports have shown that G6PD activity was increased in beta-thalassaemia carriers only when activity was calculated as IU/gHb^24^ or independently from the normalization used (either IU/gHb or U/RBC)^14^. A concurrence of microcytosis and increased number of reticulocytes might explain these findings; in this study carriers of Hb variants have increased reticulocyte numbers, which would be predictive of a lower haemolytic risk. Although the current analysis was not designed to prove this hypothesis, the data show that using a combination of spectrophotometry and flow-cytometry is a feasible approach for the study of the phenotypic interaction of G6PD deficiency and haemoglobinopathies and could be used in clinical trials performed in populations where Hb variants are very common, for example in Cambodia and Laos.

Using the flow-cytometric assay it is possible to analyse an induced haemolysis in subjects with different G6PD phenotypes treated with different drug regimens, not only by assessing haemoglobin drops but also by analyzing the actual change in the proportion of circulating G6PD deficient RBCs. The assay is also useful in studying the dynamics of haemolysis in subjects with acute malaria and with different parasitaemic loads. This will allow a better comprehension of the relationship between drug dosage and haemolysis in subjects with different G6PD variants and activity levels in subjects with virtually any haematologic picture (including anaemia of various aetiologies and abnormal haemoglobins). Field based characterization of G6PD phenotype will likely be done in the future through the use of Point-Of-Care (POC) quantitative tests (for example Biosensor machine^25^). Haemoglobin concentration might be part of the algorithm to decide whether treatment with primaquine and other 8-aminoquinolines can be used. A flow-cytometric G6PD assay with characteristics of POC is not feasible now for staining constraints (including use of sodium nitrite and potassium cyanide and incubation duration) but might be available in the near future since portable flow-cytometers already exist (for example^26^ and newer models with blue laser).

In conclusion, our data confirm that spectrophotometry and flow-cytometry are reliable techniques for the assessment of G6PD phenotype in non-anaemic subjects with normal Hb type. In anaemic subjects and in carriers of haemoglobinopathies the flow-cytometric assay is not influenced by the Hb concentration, the total number of RBCs or the reticulocyte count and can accurately estimate the percentage of G6PD deficient RBCs. The flow-cytometric assay represents a powerful tool for interpreting haemolytic risk and should be used in the future for assessment of post-treatment haemolysis in malaria patients treated with 8-aminoquinolines.

## Methods

### Human subjects research

Specimens from Thailand were collected by the Shoklo Malaria Research Unit (SMRU, Mae Sot, Tak Province) from volunteers between February and April 2014. Ethical approvals for this study were obtained from the Mahidol University Faculty of Tropical Medicine Ethics Committee (TMEC 13-017 and MO/13/096), Oxford Tropical Research Ethics Committee (OxTREC 1010-13), and the PATH Research Ethics Committee (REC SMRU1302). The protocol was also reviewed by the Tak Community Advisory Board, which is composed of representatives from the communities within the catchment area of SMRU. Volunteers who met the inclusion criteria underwent a detailed informed consent process, and provided written consent before enrolling in the study. All experiments were performed in accordance with relevant guidelines and regulations.

US donor blood specimens were obtained by Bioreclamation, Inc. (Westbury, NY) and were collected between January 2012 and January 2016 from volunteers who were at least 18 years of age, and who signed consent under institutional review board protocol by the Schulman IRB (Cincinnati, OH, USA), 2010-017 IRB. All donors were of African-American origin, presenting at a recruitment center in New York, USA.

### Specimen collection and processing

Sample collection in Thailand among 146 adult healthy volunteers was already described in Bancone *et al*., 2015; sample collection in the USA among 97 healthy volunteers of African-American heritage was already described in ref. 8. In Thailand, venous blood was analysed for complete blood count (Nihon Kohden CeltacF MEK-8222K), manual RBC morphology was assessed using Wright stain and manual reticulocyte count was assessed using New Methylene Blue. Haemoglobin variants were analysed using Capillary Electrophoresis (Capillarys2, SEBIA, France). In both the Asian and African-American samples, G6PD mutations were analysed by gene sequencing as in the paper from LaRue and colleagues^8^. The spectrophotometric assay was performed in duplicate on whole blood samples using Trinity kits (Trinity, Ireland) and the activity was calculated as IU/gHb and U/RBC using data obtained from the complete blood count. Calculation of activity per number of RBCs was suggested in the presence of hypochromic RBCs^27^. The flow-cytometry based assay was performed in duplicate according to published protocol using 5 µl of packed RBCs^9^ and was analysed using an Accuri™ C6 flow-cytometer (BD Biosciences, Singapore) with a blue laser (488 nm) on a total of 30,000 RBCs per replicate. In the Asian samples the flow-cytometry read-outs were performed on freshly stained samples and repeated on samples kept at room temperature after staining for 1–3 hours in order to assess stability of analysis overtime.

### Statistical analysis

Flow-cytometry raw data were imported into R, a statistical programming software environment^28^. The characterization of the number of bright cells, the metric used to gauge G6PD activity in the MRT using flow-cytometric techniques was done using proprietary software developed by PATH within the R environment (https://mkalnoky.shinyapps.io/MosaicG6PDflow/).

For the Asian samples only, flow-cytometry read-outs were also analysed using FlowJo software (Tree Star Inc, USA). After the exclusion of duplets on FSC-A/FSC-H dot plot, FL1 fluorescence intensity (Blue laser detection with a Band Pass 533/15 nm) was plotted to analyse distribution of fluorescence in RBCs. Deficient samples showed a single peak distribution in the low fluorescence range (left part) with often a second small peak in the high fluorescence zone which represents reticulocytes and young mature erythrocytes; G6PD normal samples usually showed a single peak distribution in the high intensity range (right part). Samples from heterozygous women showed bi-modal distribution with varying relative proportion of cells in each range. The central limit of the bi-modal distribution was used as cut-off for defining percentage of G6PD deficient and normal RBCs in all the other samples. Correlation between the Mosaic G6PD Flow Tool and the FlowJo analysis was analysed by linear regression.

Differences in haematologic parameters among groups were analysed by Chi square or ANOVA. Anaemia was defined as Hb concentration ≤ 11.5 g/dL^29^. Pearson’s coefficient was used to analyse the correlation between enzymatic activities as assessed by the different assays. The comparison of G6PD activity between spectrophotometry and flow-cytometry in heterozygous women was performed in the following way: the activity by spectrophotometry (calculated as IU/gHb or U/RBCs) was expressed as a percentage of the population median and compared with the actual percentage of normal RBCs as counted by the flow cytometer using Bland-Altman analysis. Furthermore the residuals from a best linear fit between spectrophotometry and flow-cytometry were plotted against their mean to inspect for agreement between the two assays^30^.

### Data Availability

The datasets generated during and/or analysed during the current study are available at https://dataverse.harvard.edu/dataverse/G6PD-flow-cytometric or from the corresponding author.

## Electronic supplementary material

Supplementary Information

## References

[1] Cappellini, M. D. & Fiorelli G. Glucose-6-phosphate dehydrogenase deficiency. *Lancet.* **371(9606)** 64–74 doi:10.1016/S0140-6736(08)60073-2. Review 5 Jan 2008. 10.1016/S0140-6736(08)60073-2 18177777

[2] Chu, G. S. *et al*. Haemolysis in G6PD Heterozygous Females Treated with Primaquine for Plasmodium vivax Malaria: A Nested Cohort in a Trial of Radical Curative Regimens. *PLoS Med.* **14(2)**: e1002224. doi:10.1371/journal.pmed.1002224. eCollection 7 Feb 2017. 10.1371/journal.pmed.1002224 PMC5295665 28170391

[3] Beutler E (1977). International Committee for Standardization in Haematology: recommended methods for red-cell enzyme analysis. British journal of haematology.

[4] Brewer GJ, Tarlov AR, Alving AS (1960). Methaemoglobin reduction test: a new, simple, *in vitro* test for identifying primaquine-sensitivity. Bulletin of the World Health Organization.

[5] Fairbanks VF, Lampe LT (1968). A tetrazolium-linked cytochemical method for estimation of glucose-6-phosphate dehydrogenase activity in individual erythrocytes: applications in the study of heterozygotes for glucose-6-phosphate dehydrogenase deficiency. Blood.

[6] Van Noorden CJ, Vogels IM (1985). A sensitive cytochemical staining method for glucose-6-phosphate dehydrogenase activity in individual erythrocytes. II. Further improvements of the staining procedure and some observations with glucose-6-phosphate dehydrogenase deficiency. British journal of haematology.

[7] Peters AL, Van Noorden CJ (2009). Glucose-6-phosphate dehydrogenase deficiency and malaria: cytochemical detection of heterozygous G6PD deficiency in women. The journal of histochemistry and cytochemistry: official journal of the Histochemistry Society.

[8] LaRue N (2014). Comparison of quantitative and qualitative tests for glucose-6-phosphate dehydrogenase deficiency. The American journal of tropical medicine and hygiene.

[9] Shah SS (2012). A novel cytofluorometric assay for the detection and quantification of glucose-6-phosphate dehydrogenase deficiency. Scientific reports.

[10] Gall JC, Brewer GJ, Dern RJ (1965). Studies of Glucose-6-Phosphate Dehydrogenase Activity of Individual Erythrocytes: The Methemoglobin-Elution Test for Identification of Females Heterozygous for G6PD Deficiency. American journal of human genetics.

[11] Stamatoyannopoulos G, Papayannopoulou T, Bakopoulos C, Motulsky AG (1967). Detection of glucose-6-phosphate dehydrogenase deficient heterozygotes. Blood.

[12] Vives-Corrons JL, Kuhl W, Pujades MA, Beutler E (1990). Molecular genetics of the glucose-6-phosphate dehydrogenase (G6PD) Mediterranean variant and description of a new G6PD mutant, G6PD Andalus1361A. American journal of human genetics.

[13] Vogels IM (1986). Cytochemical determination of heterozygous glucose-6-phosphate dehydrogenase deficiency in erythrocytes. British journal of haematology.

[14] Sanna G (1980). Interaction between the glucose-6-phosphate dehydrogenase deficiency and thalassaemia genes at phenotype level. British journal of haematology.

[15] Kahn M (2015). Maintaining Specimen Integrity for G6PD Screening by Cytofluorometric Assays. The journal of histochemistry and cytochemistry: official journal of the Histochemistry Society.

[16] Ellis D, Sewell CE, Skinner LG (1956). Reticulocyte enzymes and protein synthesis. Nature.

[17] Srivastava A, Evans KJ, Sexton AE, Schofield L, Creek DJ (2017). Metabolomics-Based Elucidation of Active Metabolic Pathways in Erythrocytes and HSC-Derived Reticulocytes. Journal of proteome research.

[18] Williams TN, Weatherall DJ (2012). World distribution, population genetics, and health burden of the hemoglobinopathies. Cold Spring Harbor perspectives in medicine.

[19] Weatherall DJ, Clegg JB (2001). Inherited haemoglobin disorders: an increasing global health problem. Bulletin of the World Health Organization.

[20] Chen Q (2012). A transgenic mouse model expressing exclusively human hemoglobin E: indications of a mild oxidative stress. Blood cells, molecules & diseases.

[21] Voskou S, Aslan M, Fanis P, Phylactides M, Kleanthous M (2015). Oxidative stress in beta-thalassaemia and sickle cell disease. Redox biology.

[22] Bernini L (1964). Survival of 51 Cr-Labelled Red Cells in Subjects with Thalassaemia-Trait or G6pd Deficiency or Both Abnormalities. British journal of haematology.

[23] Carcassi UE (1974). The interaction between beta-thalassemia, G-6-PD deficiency, and favism. Annals of the New York Academy of Sciences.

[24] Piomelli S, Siniscalco M (1969). The haematological effects of glucose-6-phosphate dehydrogenase deficiency and thalassaemia trait: interaction between the two genes at the phenotype level. British journal of haematology.

[25] Ley B (2017). A Comparison of Three Quantitative Methods to Estimate G6PD Activity in the Chittagong Hill Tracts, Bangladesh. PloS one.

[26] Dittami GM, Sethi M, Rabbitt RD, Ayliffe HE (2012). Determination of mammalian cell counts, cell size and cell health using the Moxi Z mini automated cell counter. Journal of visualized experiments: JoVE.

[27] Group WS (1967). Standardization of procedures for the study of glucose-6-phosphate dehydrogenase. Report of a WHO Scientific Group. World Health Organization technical report series.

[28] R: A language and environment for statistical computing. R Foundation for Statistical Computing (Vienna, Austria, 2013).

[29] Beutler E, Waalen J (2006). The definition of anemia: what is the lower limit of normal of the blood hemoglobin concentration?. Blood.

[30] Giavarina D (2015). Understanding Bland Altman analysis. Biochemia medica.

