# Supplementary material for: The G6PD flow-cytometric assay is a reliable tool for diagnosis of G6PD deficiency in women and anaemic subjects
Source: Sci Rep. 2017 Aug 29;7:9822. doi: 10.1038/s41598-017-10045-2 (PMC5575121; doi:10.1038/s41598-017-10045-2)
Supplement: Supplementary file 1 — Supplementary Information [file 41598_2017_10045_MOESM1_ESM.pdf]

Supplementary Information for
"The G6PD flow-cytometric assay is a reliable tool for diagnosis of G6PD deficiency in women and anaemic subjects"

Germana Bancone[1,5*], Michael Kalnoky[2], Cindy S. Chu[1,5], Nongnud Chowwiwat[1], Maria Kahn[2], Benoit Malleret[3,4], Pornpimon Wilaisrisak[1], Laurent Rénia[3], Gonzalo J. Domingo[2] and Francois Nosten[1,5]

[1] Shoklo Malaria Research Unit, Mahidol–Oxford Tropical Medicine Research Unit, Faculty of Tropical Medicine, Mahidol University, Mae Sot, Thailand
[2] Diagnostics Program, PATH, Seattle, USA
[3] Singapore Immunology network (SIgN), A*STAR, 8A Biomedical Grove, Singapore 138648, Singapore.
[4]Department of Microbiology and Immunology, Yong Loo Lin School of Medicine, National University of Singapore, National University Health System, 5 Science Drive 2, Blk MD4, Level 3, Singapore 117597, Singapore.
[5] Centre for Tropical Medicine, Nuffield Department of Medicine, University of Oxford, Oxford, United Kingdom
* E-mail: germana@tropmedres.ac

Table S1. Summary of study genotypes for African-American and Asian cohorts as determined by DNA sequencing

Study site	Gender	Genotype	Mutation	Amino Acid substitution	N
African-American	Male	Hemizygote	Wild type	none	23
African-American	Male	Hemizygote	A+	N126D	6
African-American	Male	Hemizygote	A-	N126D and V68M	18
African-American	Female	Homozygote	Wild type	none	18
African-American	Female	Heterozygote	A+	N126D	14
African-American	Female	Heterozygote	A-	N126D and V68M	18
Asian	Male	Hemizygote	Wild type	none	26
Asian	Male	Hemizygote	Viangchan	G291A	1
Asian	Male	Hemizygote	Mahidol	G163S	23
Asian	Female	Homozygote	Wild type	none	20
Asian	Female	Heterozygote	Mahidol	G163S	55
Asian	Female	Heterozygote	Kaiping	R463H	1
Asian	Female	Heterozygote	Shoklo	I234T	1
Asian	Female	Heterozygote	Mediterranean	S188F	1
Asian	Female	Homozygote	Mahidol	G163S	17
Asian	Female	Homozygote	Orissa and Mahidol	C44G and G163S	1

Figure S1. Post-staining haemolysis of G6PD deficient RBCs in samples with different levels of enzymatic activity (only Asian sample)

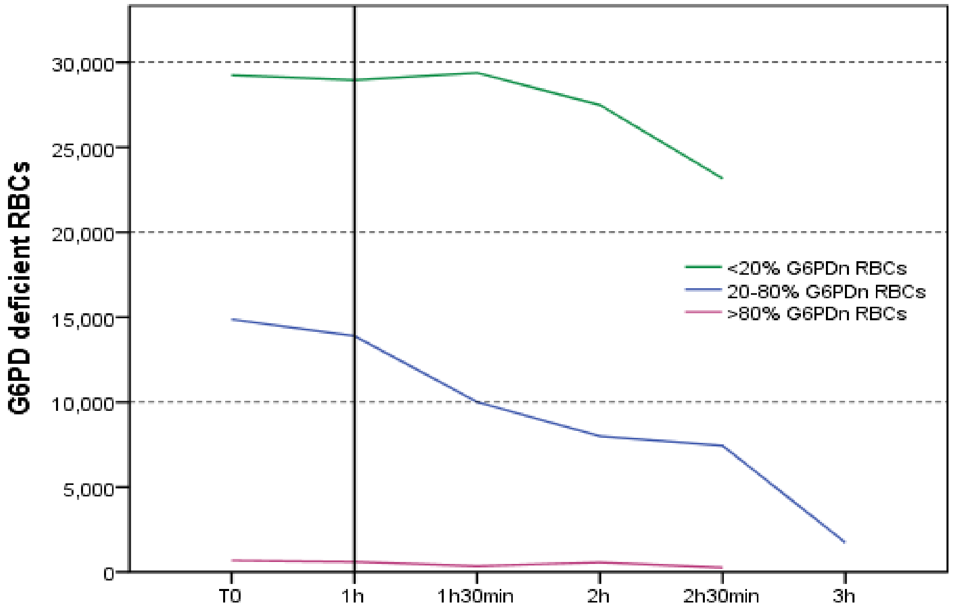

The green line represents G6PD deficient samples (<20% bright cells), the blue line represents intermediate samples (20-80% bright cells) and the red line represents normal samples (>80% bright cells).

Figure S2. Histogram of fluorescence on FL-1 channel in two G6PD Mahidol heterozygous samples analyzed right after staining (t0), 1 hour post-staining (t1h) and 2 hours post-staining (t2h)

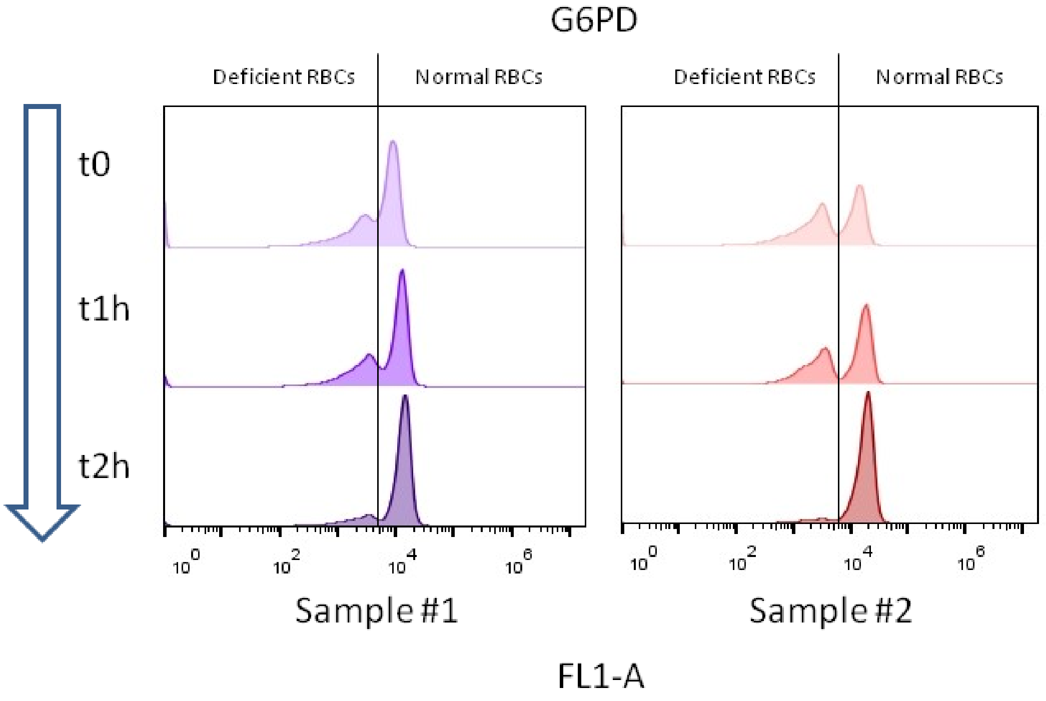

Table S2. Changes in absolute number of deficient RBCs over time following staining for flow cytometric assay in the Asian sample

PHENOTYPE		Δ at T=1h	Δ at T=1h30min	Δ at T=2h	Δ at T=2h30min	Δ at T=3h
<20% G6PDn RBCs	Mean	-259	-42	-1467	-6257	
	N	76	8	45	6	
	Std. Deviation	1740	252	3637	8878	
20-80% G6PDn RBCs	Mean	-1175	-4080	-6431	-7103	-10684
	N	85	14	49	12	4
	Std. Deviation	1267	2419	4013	3332	5564
>80% G6PDn RBCs	Mean	-88	-442	-205	138	
	N	77	9	62	2	
	Std. Deviation	339	276	469	9	
Total	Mean	-531	-1982	-2525	-6125	-10684
	N	238	31	156	20	4
	Std. Deviation	1342	2518	4016	5649	5564

Table S3. Statistical calculations for comparisons of reticulocyte and MCV among subjects with different G6PD and Hb status

Statistical Calculations to Compare Reticulocytes and MCV for different G6PD zygosities from normal, mutated and abnormal Hb values				
**Fig 1 A**				
**Differences in MCV Normal Hb**	**p value**		**Differences in MCV Mutated Hb**	**p value**
heterozygous vs. hemi/homozygous	0.036		heterozygous vs. hemi/homozygous	0.992
WT vs. hemi/homozygous	0.005		WT vs. hemi/homozygous	0.895
WT vs. heterozygous	0.636		WT vs. heterozygous	0.959
**Fig 1 B**				
**Differences in Reticulocyte Normal Hb**	**p value**		**Differences in Reticulocyte Mutated Hb**	**p value**
heterozygous vs. hemi/homozygous	0.914		heterozygous vs. hemi/homozygous	0.246
WT vs. hemi/homozygous	0.002		WT vs. hemi/homozygous	0.220
WT vs. heterozygous	0.000		WT vs. heterozygous	0.971
**Fig 1 C**				
**Differences in Reticulocyte Normal Hb**	**p value**		**Differences in Reticulocyte Abnormal Hb**	**p value**
heterozygous vs. hemi/homozygous	0.771		heterozygous vs. hemi/homozygous	0.255
WT vs. hemi/homozygous	0.002		WT vs. hemi/homozygous	0.025
WT vs. heterozygous	0.014		WT vs. heterozygous	0.207

Statistical Calculations to compare Reticulocytes and MCV for normal, abnormal and mutated Hb values within each G6PD zygosity	
**Fig 1 A Differences in MCV**	**p value**
hemi/homozygous Hb Normal vs. hemi/homozygous Hb Mutated	0.000
heterozygous Hb Mutated vs. hemi/homozygous Hb Mutated	1.000
heterozygous Hb Normal vs. hemi/homozygous Hb Mutated	0.000
WT Hb Mutated vs. hemi/homozygous Hb Mutated	0.959
WT Hb Normal vs. hemi/homozygous Hb Mutated	0.000
heterozygous Hb Mutated vs. hemi/homozygous Hb Normal	0.000
heterozygous Hb Normal vs. hemi/homozygous Hb Normal	0.177
WT Hb Mutated vs. hemi/homozygous Hb Normal	0.000
WT Hb Normal vs. hemi/homozygous Hb Normal	0.037
heterozygous Hb Normal vs. heterozygous Hb Mutated	0.000
WT Hb Mutated vs. heterozygous Hb Mutated	0.996
WT Hb Normal vs. heterozygous Hb Mutated	0.000
WT Hb Mutated vs. heterozygous Hb Normal	0.000
WT Hb Normal vs. heterozygous Hb Normal	0.956
WT Hb Normal vs. WT Hb Mutated	0.000
**Fig 1 B Differences in Reticulocytes**	**p value**
hemi/homozygous Hb Normal vs. hemi/homozygous Hb Mutated	0.002
heterozygous Hb Mutated vs. hemi/homozygous Hb Mutated	0.078
heterozygous Hb Normal vs. hemi/homozygous Hb Mutated	0.003
WT Hb Mutated vs. hemi/homozygous Hb Mutated	0.055
WT Hb Normal vs. hemi/homozygous Hb Mutated	0.000
heterozygous Hb Mutated vs. hemi/homozygous Hb Normal	1.000
heterozygous Hb Normal vs. hemi/homozygous Hb Normal	0.999
WT Hb Mutated vs. hemi/homozygous Hb Normal	1.000
WT Hb Normal vs. hemi/homozygous Hb Normal	0.013
heterozygous Hb Normal vs. heterozygous Hb Mutated	1.000
WT Hb Mutated vs. heterozygous Hb Mutated	0.999
WT Hb Normal vs. heterozygous Hb Mutated	0.961
WT Hb Mutated vs. heterozygous Hb Normal	1.000
WT Hb Normal vs. heterozygous Hb Normal	0.001
WT Hb Normal vs. WT Hb Mutated	0.515
**Fig 1 C Differences in Reticulocytes**	**p value**
hemi/homozygous Hb Normal vs. hemi/homozygous Hb Abnormal	0.001
heterozygous Hb Abnormal vs. hemi/homozygous Hb Abnormal	0.245
heterozygous Hb Normal vs. hemi/homozygous Hb Abnormal	0.000
WT Hb Abnormal vs. hemi/homozygous Hb Abnormal	0.003
WT Hb Normal vs. hemi/homozygous Hb Abnormal	0.000
heterozygous Hb Abnormal vs. hemi/homozygous Hb Normal	0.072

heterozygous Hb Normal vs. hemi/homozygous Hb Normal	0.991
WT Hb Abnormal vs. hemi/homozygous Hb Normal	0.997
WT Hb Normal vs. hemi/homozygous Hb Normal	0.033
heterozygous Hb Normal vs. heterozygous Hb Abnormal	0.016
WT Hb Abnormal vs. heterozygous Hb Abnormal	0.174
WT Hb Normal vs. heterozygous Hb Abnormal	0.000
WT Hb Abnormal vs. heterozygous Hb Normal	1.000
WT Hb Normal vs. heterozygous Hb Normal	0.133
WT Hb Normal vs. WT Hb Abnormal	0.709
